# *Notes from the Field:* Opioid-Involved Overdose Deaths with Fentanyl or Fentanyl Analogs Detected — 28 States and the District of Columbia, July 2016–December 2018

**DOI:** 10.15585/mmwr.mm6910a4

**Published:** 2020-03-13

**Authors:** Julie O’Donnell, R. Matt Gladden, Bruce A. Goldberger, Christine L. Mattson, Mbabazi Kariisa

**Affiliations:** ^1^Division of Overdose Prevention, National Center for Injury Prevention and Control, CDC; ^2^Forensic Medicine Division, Department of Pathology, Immunology and Laboratory Medicine, College of Medicine, University of Florida, Gainesville, Florida.

Approximately two thirds of the 70,237 U.S. drug overdose deaths reported in 2017 involved opioids (*1*). Since 2013, opioid-involved overdose deaths involving illicitly manufactured fentanyl has sharply increased (*1*,*2*). Fentanyl analogs are structurally similar to fentanyl but vary in potency, are primarily illicitly distributed, and require specific postmortem toxicology testing for detection.* Deaths involving fentanyl analogs, particularly carfentanil, increased in 10 states during 2016–2017 and often co-occurred with fentanyl (*3*). CDC funded 32 states and the District of Columbia (DC) to enhance postmortem toxicology testing and abstract data from death certificates and medical examiner and coroner reports on opioid-involved overdose deaths of unintentional and undetermined intent through the State Unintentional Drug Overdose Reporting System (SUDORS).^†^ Twelve states have collected data since July 2016, and an additional 20 states and DC began collecting data in July 2017 as part of a rapid expansion of SUDORS. This analysis 1) reports rapid changes in opioid-involved overdose deaths with fentanyl^§^ and fentanyl analogs detected during July 2016–December 2018 among 10 states with available SUDORS data^¶^ and 2) provides a description of the most recent data on deaths with fentanyl and fentanyl analogs detected among 28 states and DC.** Tracking specific drugs involved in overdose deaths is critical because the risk for overdose for fentanyl and fentanyl analogs varies substantially. There are considerable differences in potency, dose, purity, and co-use patterns among drug products.^††^

During July 2016–December 2018, a total of 26,104 opioid-involved overdose deaths were reported in the 10 states, including 5,083 (19.5%) for which at least one fentanyl analog was detected. Among these deaths, more than 15 different fentanyl analogs were identified, with more than one analog detected in some deaths; the five most commonly detected were acetylfentanyl (2,178 deaths; 8.3% of opioid-involved overdose deaths), carfentanil (1,724; 6.6%), furanylfentanyl (497; 1.9%), cyclopropylfentanyl (371; 1.4%), and acrylfentanyl (353; 1.4%). Deaths associated with carfentanil, furanylfentanyl, cyclopropylfentanyl, and acrylfentanyl peaked at different times (Figure). Deaths with carfentanil detected peaked twice, in September 2016 (87 deaths) and in April 2017 (235). Deaths with furanylfentanyl detected peaked in January 2017 (100), those with acrylfentanyl detected peaked in February 2017 (122), and those with cyclopropylfentanyl detected peaked in September 2017 (49). Deaths with these four analogs detected decreased to fewer than five each by December 2018. In contrast, acetylfentanyl was increasingly detected in opioid-involved overdose deaths over time, reaching 179 in December 2018. Fentanyl deaths increased by 25.8%, from 3,086 during July–December 2016 to 3,881 during July–December 2018.

**FIGURE:**
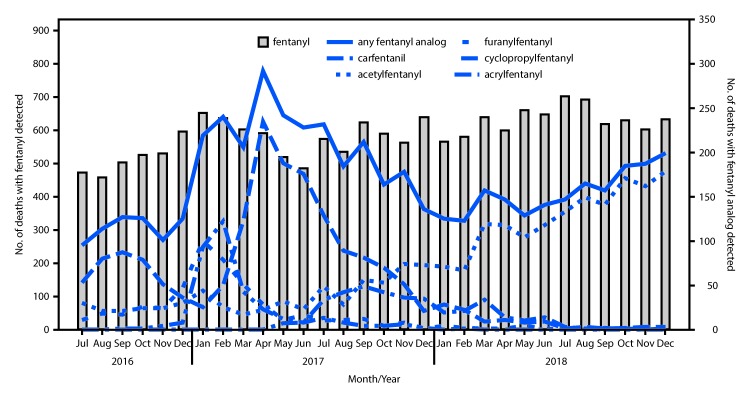
Number of opioid-involved overdose deaths with fentanyl or the five most common fentanyl analogs detected — State Unintentional Drug Overdose Reporting System, 10 states,* July 2016–December 2018 * Kentucky, Maine, Massachusetts, Missouri, New Hampshire, New Mexico, Ohio, Oklahoma, Rhode Island, and Wisconsin.

During July–December 2018, in 28 states and DC, one or more fentanyl analogs were detected in 2,824 (19.4%) of 14,571 opioid-involved overdose deaths. The most commonly detected analogs during that period were acetylfentanyl (2,363; 16.2% of opioid-involved overdose deaths), a combined group of “fluorofentanyls”^§§^ (269; 1.8%), butyrylfentanyl (86; 0.6%), methoxyacetylfentanyl (85; 0.6%), and valerylfentanyl (71; 0.5%). Excluding acetylfentanyl, all other fentanyl analogs were detected in <5% of opioid-involved overdose deaths. Fentanyl was detected in 73.9% of opioid-involved overdose deaths.

The declines in overdose deaths with the fentanyl analogs carfentanil, furanylfentanyl, acrylfentanyl, and cyclopropylfentanyl detected contributed to previously reported declines in opioid-involved overdose deaths during 2018 among 25 states, even as deaths with fentanyl detected increased over time (*4*). This suggests a shift away from illicit fentanyl analog distribution to distribution of illicitly manufactured fentanyl.^¶¶^ Increased acetylfentanyl detection must be interpreted cautiously. Specifically, acetylfentanyl might be a byproduct or contaminant in illicitly manufactured fentanyl products rather than being intentionally distributed*** (*5*). Although fentanyl analog–associated deaths occurred infrequently by the end of 2018, recent reports indicate that fentanyl analogs might reemerge. An Ohio county reported sharp increases in carfentanil-involved deaths in 2019, and Ontario, Canada, issued a 2019 alert reporting increases in carfentanil-involved overdose deaths.^†††^ Timely toxicologic surveillance is critical to accurately detect opioid-involved overdose deaths and, in turn, to inform interventions that could mitigate health consequences of rapid illicit drug market changes.

## Footnotes

* Fentanyl analogs, also known as fentanyl-related substances, are synthetic opioids similar in chemical structure to fentanyl but modified to produce distinct substances. Fentanyl analogs vary in potency, with some more potent than fentanyl (e.g., carfentanil and 3-methylfentanyl) and others with potency similar to or less than that of fentanyl (e.g., acetylfentanyl and furanylfentanyl). https://www.deadiversion.usdoj.gov/drug_chem_info/frs.pdf.

† SUDORS funded 32 states and DC to collect detailed information on toxicology, route of administration, and other risk factors that might be associated with a fatal opioid-involved overdose. SUDORS was part of CDC’s Enhanced State Opioid Overdose Surveillance program. SUDORS data are reported to CDC twice yearly, documenting deaths that occur during the first half (January–June) and the second half (July–December) of each calendar year. Data for this report were downloaded on October 30, 2019, and might differ from earlier or future reports because states continually update death data and investigations of suspected drug overdose deaths might involve lengthy investigations. https://www.cdc.gov/drugoverdose/foa/state-opioid-mm.html.

§ A positive test for fentanyl indicates the use of prescription fentanyl, illicitly manufactured fentanyl, or both. Previous reports have established that most overdose deaths testing positive for fentanyl are related to the use of illicitly manufactured fentanyl. https://www.cdc.gov/mmwr/volumes/67/wr/mm6734a2.htm.

¶ The 10 states included are Kentucky, Maine, Massachusetts, Missouri, New Hampshire, New Mexico, Ohio, Oklahoma, Rhode Island, and Wisconsin. Data were considered validated if data quality checking processes identified no substantial issues with the number of deaths entered (e.g., unable to obtain data from a portion of the state) or quality of data entered on required SUDORS variables.

** The 29 jurisdictions included are Alaska, Connecticut, DC, Delaware, Georgia, Illinois, Indiana, Kentucky, Maine, Maryland, Massachusetts, Minnesota, Missouri, Nevada, New Hampshire, New Jersey, New Mexico, North Carolina, Ohio, Oklahoma, Pennsylvania, Rhode Island, Tennessee, Utah, Vermont, Virginia, Washington, West Virginia, and Wisconsin.

†† https://www.cdc.gov/drugoverdose/data/fentanyl.html.

§§ Because of the rapid emergence of fentanyl analogs in overdose deaths, fentanyl analogs detected on forensic toxicology laboratory tests were entered into SUDORS using a dropdown menu (preferred) or direct entry into a text field for substances not in the menu. Consequently, certain analogs cannot be distinguished in the current data and have therefore been combined into a group of analogs with “fluoro” appearing in the name: fluorobutyrylfentanyl, 4/para-fluorobutyrylfentanyl, fluoroisobutyrylfentanyl, and 4/para-fluoroisobutyrylfentanyl.

¶¶ With few exceptions, fentanyl analogs are illicitly distributed because they do not have a legitimate medical use in humans. The three fentanyl analogs with legitimate human medical use are alfentanil, remifentanil, and sufentanil.

*** For nearly all (98.6%) deaths with acetylfentanyl detected during July 2016–December 2018, fentanyl was also detected

††† http://medicalexaminer.cuyahogacounty.us/pdf_medicalexaminer/en-US/HeroinFentanylReports/011920-HeroinFentanylReport.pdf; https://www.hamilton.ca/public-health/reporting/hamilton-opioid-information-system.
